# SARS-CoV-2 reinfection in two patients who have recovered from COVID-19

**DOI:** 10.1093/pcmedi/pbaa031

**Published:** 2020-09-04

**Authors:** Kang Zhang, Johnson Yiu-Nam Lau, Lei Yang, Zhen-Guo Ma

**Affiliations:** Faculty of Medicine and University Hospital, Macau University of Science and Technology, Macau SAR, China; Department of Applied Biology and Chemical Technology, Hong Kong Polytechnic University, Hong Kong SAR, China; The First Affiliated Hospital, Sun Yat-sen University, Guangzhou 510080, China; Renmin Hospital of Wuhan University, Wuhan 430060, China

**Keywords:** asymptomatic carriers, reinfection, COVID-19

**Dear Editor,**

The duration of immune protection against SAR-CoV-2 reinfection in patients who have recovered from COVID-19 is a critical unanswered question. To determine the long-term clinical and virologic outcome in patients who have recovered from COVID-19, we prospectively followed 220 patients with COVID-19 admitted to Renmin Hospital of Wuhan University between 15 January and 15 February 2020 who recovered and were followed up until 15 July 2020. Throat swab SARS-CoV-2 RNA was tested regularly during hospitalization and follow-up using reverse transcription polymerase chain reaction (RT-PCR).^1^

Of the 220 patients, 193 patients recovered from COVID-19 infection and their throat swab viral RNA remained negative during follow-up. For the remaining 27 patients with positive throat swab viral RNA during follow-up, we identified three different virologic patterns.

First, 16 patients (M:F 5:11, age 24–77) had positive throat swab viral RNA (usually with a weak signal, + out of +++ signal) during their first admission, then were discharged home when their viral RNA was negative. After an average of 3.7 days (range 1–16 days), the follow-up viral RNA was positive again [usually also with a weak signal (+)] for a period of 5–57 days before turning negative again. As all patients with positive viral RNA were required to stay in the hospital, the first admission was for 4–56 days and the second admission was for 5–57 days. COVID-19 disease severity was assessed based on the classification recommended by the Chinese Health Authority [7^th^ edition; mild—asymptomatic/relatively asymptomatic with no chest x-ray film (CXR)/chest CT changes; moderate—fever and/or respiratory symptoms and/or with radiological evidence of pneumonia (can be relatively asymptomatic but with radiologic changes); severe—severe respiratory symptoms with oxygen saturation of <93%, drop in arterial oxygen tension or CXR/chest CT showing ≥50% lung areas involved; and ICU admission—respiratory distress requiring mechanical ventilation or organ failures requiring ICU care]. For the first submission, the disease severity was classified as mild 1, moderate 11, severe 4, and ICU 0; and for the second submission, the severity was classified as mild 3, moderate 13, and severe and ICU admission 0. All 16 patients were symptomatic on their first submission, but 9 of the 16 patients were relatively asymptomatic during their second submission. These patients might have had intermittent viral RNA positivity during their recovery; however, the observation that the second admission covered up to 57 days of viral RNA positivity, with 9/16 patients being relatively asymptomatic highlights the importance of virologic follow-up of recovered patients to identify any that revert to having positive viral RNA so as to reduce the pool of infection sources.

Second, 9 patients (M:F 2:7, age 22–78) showed strong signals for throat swab viral RNA based on RT-PCR (majority +++), which turned negative for 2–13 days, and then turned strongly positive (+++) again for 7–28 days. Their disease severity was mild 0, moderate 7, severe 2, and ICU 0 for their first admission (5–35 days); and mild 3, moderate 6, severe 0, and ICU 0 for their second admission (7–28 days). During the first admission, 1/9 patients was relatively asymptomatic and during the second admission, 6/9 patients were relatively asymptomatic. Again, these patients could serve as potential source of infection. Whether these big fluctuations of viral RNA in their throat swabs represented viral RNA titer variations or whether these 9 subjects displayed viral reactivation or new viral infections were difficult to determine clinically.

Third, in 2 patients (both F and both 33 years old), their first admission showed disease severity of 1 moderate and 1 severe, and they were in hospital for 16 and 38 days respectively. Both recovered clinically and were discharged with negative throat swab viral RNA. They were clinically well and negative for viral RNA during follow-up for 43 and 58 days. Then, they became positive for throat viral RNA again, and both had a return to moderate disease severity during the second admission (66 and 75 days). Before the second admission, both patients also showed reductions in IgG anti-SARS-CoV-2 antibody,^1^ with one being seronegative and one weak positive before throat swab viral RNA was positive again. During the second admission, both patients showed increases in their IgG anti-SARS-CoV-2 titers, and one patient also showed renewed reactivity for IgM anti-SARS-CoV-2. As both had recovered clinically and had negative throat swab viral RNA for 43–58 days before they became sick again with positive throat swab viral RNA together with an increase in IgG antibody (with one also showing IgM again), they were considered as having SARS-CoV-2 reinfection. Whether the low/absent IgG before the reinfection predisposed these two patients to reinfection requires further clarification in a bigger cohort of follow-up subjects. However, the observation of these reinfections suggests that the concept of herd immunity protection may not be applicable in at least a small proportion of patients who have recovered from COVID-19.

These observations highlighted, first, the importance of following up with patients who have recovered from COVID-19 to ensure that we can identify the small percentage of patients who may revert to positive viral RNA. As some of the patients who reverted to positive viral PCR were relatively asymptomatic, they could still serve as sources for new infections.^2^ Second, both clinical and virologic profiles indicated that two patients in our cohort had SARS-CoV-2 reinfection, suggesting that the herd immunity approach may not protect all patients who have recovered.
